# Variability in bacterial flagella re-growth patterns after breakage

**DOI:** 10.1038/s41598-017-01302-5

**Published:** 2017-04-28

**Authors:** Guillaume Paradis, Fabienne F. V. Chevance, Willisa Liou, Thibaud T. Renault, Kelly T. Hughes, Simon Rainville, Marc Erhardt

**Affiliations:** 1 Department of Physics, Engineering Physics and Optics and Centre of Optics, Photonics and Lasers, Laval University, Quebec City, Quebec Canada; 2 Department of Biology, University of Utah, Salt Lake City, Utah 84112 USA; 3 Helmholtz Centre for Infection Research, 38124 Braunschweig, Germany

## Abstract

Many bacteria swim through liquids or crawl on surfaces by rotating long appendages called flagella. Flagellar filaments are assembled from thousands of subunits that are exported through a narrow secretion channel and polymerize beneath a capping scaffold at the tip of the growing filament. The assembly of a flagellum uses a significant proportion of the biosynthetic capacities of the cell with each filament constituting ~1% of the total cell protein. Here, we addressed a significant question whether a flagellar filament can form a new cap and resume growth after breakage. Re-growth of broken filaments was visualized using sequential 3-color fluorescent labeling of filaments after mechanical shearing. Differential electron microscopy revealed the formation of new cap structures on broken filaments that re-grew. Flagellar filaments are therefore able to re-grow if broken by mechanical shearing forces, which are expected to occur frequently in nature. In contrast, no re-growth was observed on filaments that had been broken using ultrashort laser pulses, a technique allowing for very local damage to individual filaments. We thus conclude that assembly of a new cap at the tip of a broken filament depends on how the filament was broken.

## Introduction

The flagellum of enteric bacteria consists of three main structural parts: (i) a basal body complex that spans the periplasmic space between the inner and outer membranes and is embedded in the cell wall; (ii) an external, flexible linking structure (the hook) and (iii) a rigid, helical filament made of several thousand flagellin subunits^1, 2^. The basal body complex harbors a flagellum-specific protein export machine^2^. This flagellar-specific type-III secretion system exports most extra-cytoplasmic building blocks of the flagellum in a proton motive force (PMF) dependent manner^3, 4^. After injection into a narrow secretion channel within the flagellar structure (~2 nm diameter), the substrates travel diffusively to the tip of the growing flagellum and self-assemble with the help of capping scaffold proteins^2, 5, 6^. The flagellar filament is connected to the hook-basal body (HBB) structure via two hook-associated proteins (FlgK, FlgL or HAP1, HAP3) and polymerization of filament subunits (flagellin, FliC or FljB in *Salmonella enterica*) requires the presence of a cap protein scaffold at the filament tip (FliD or HAP2)^7, 8^.

In *Salmonella enterica*, expression of flagellar genes is temporally coupled to the assembly state of the flagellum and can be ordered into a transcriptional hierarchy of three promoter classes^9, 10^. The flagellar master regulatory complex FlhDC is expressed from the Class 1 promoter and directs σ^70^-RNA polymerase to transcribe from Class 2 promoters. Gene products expressed from Class 2 promoters include the components of the HBB complex, as well as regulatory proteins, e.g. the flagellar-specific, alternative σ^28^ factor and its cognate anti-σ factor FlgM. The completion of the HBB complex results in a switch in secretion-substrate specificity within the type-III secretion apparatus from secretion of early (HBB-type) substrates to the secretion of late (filament-type) substrates. After the switch in secretion specificity, FlgM is secreted as a late substrate thus freeing σ^28^ to activate transcription from Class 3 promoters in response to HBB completion^11^. Class 3 gene products are needed for completion of the flagellum (e.g. the filament subunits, the filament cap, the motor-force generators) and the chemosensory system. Thus, by secreting FlgM protein after the secretion specificity switch, the cell ensures that genes needed after HBB completion are only expressed after a functional HBB structure has been assembled, onto which σ^28^-dependent gene products such as the filament subunits can polymerize.

The flagellar filament consists of several thousand subunits of flagellin and grows to lengths up to 20 µm. It is presumed that shearing of flagellar filaments occurs in nature, however it is not clear if a sheared flagellum can re-grow^12^. A sheared flagellum would need to re-assemble the filament cap structure, as the original cap would have been lost by the shearing event. In *Salmonella enterica*, the filament cap gene (*fliD*) is transcribed from both Class 2 and Class 3 promoters^13^. The FliD cap protein is expressed from its Class 2 promoter prior to HBB completion. After the switch in substrate secretion specificity, FliD is secreted simultaneously with FlgM allowing for an efficient transition to filament assembly. Thus, the FliD cap does not compete with flagellin for secretion prior to initiation of flagellin gene expression. FliD is likely expressed from its Class 3 promoter in the case of shearing events, which allows the formation of a new cap on the tip of the broken filament and thus re-growth of a sheared filament. In order to determine if flagellar filaments can re-grow, Rosu and Hughes analyzed the dynamics of Class 3 gene expression after flagellar shearing in *Salmonella enterica* ^12^. FlgM is constantly secreted during flagellar growth. The rate of flagellar substrate export decreases with the length of the filament structure^6, 14^. Thus, shearing of a filament might result in a sudden increase in the rate of FlgM secretion and a subsequent burst of Class 3 gene expression. However, the levels of intracellular FlgM and Class 3 gene expression remained unchanged after flagellar shearing^12^.

In a recent study, Turner and colleagues used fluorescent bi-color labeling of flagellar filaments to measure filament growth in *Escherichia coli* in a population-approach^15^. Differential fluorescent labeling of flagellar filaments allowed the authors to distinguish the growth of new filament segments from previously grown parts of the same filament. Turner *et al*. used viscous shearing forces to break flagellar filaments of *E. coli* and compared the filament lengths distributions of a sheared subpopulation with one that had not been sheared. The authors concluded that broken flagellar filaments of *E. coli* continued to grow. However, a caveat of their experiment was the inability to distinguish re-growth of sheared filaments from continued growth of nascent, short filaments that were not broken.

In the present study, we used three independent techniques to unambiguously determine whether individual flagellar filaments re-grow after being damaged. We demonstrate using 3-color differential fluorescent labeling of flagellar filaments that flagellar filaments are indeed able to re-grow after breakage by mechanical shearing forces. We further visualized, for the first time, the formation of new cap structures on broken filaments using differential electron microscopy. Finally, using again fluorescent labeling, we monitored the growth of individual filaments that were broken one by one with ultrashort laser pulses. Filaments broken with this method were not observed to re-grow, in contrast to the mechanically sheared ones. Thus, we conclude that re-growth of flagellar filaments depends on the method of breakage.

## Results

The differential fluorescent dual-color labeling of flagellar filaments pioneered by Turner and colleagues^15^ was modified by adding a third labeling step^6^, which allowed us to unequivocally determine if a broken filament re-grew. Strain EM4067 harbored the flagellar master regulatory operon under control of an anhydrotetracycline (AnTc) inducible promoter (P_*tetA*_-*flhDC*) for synchronized production of flagellar basal bodies, and a mutant flagellin *fliC* with a single cysteine amino acid substitution under control of an arabinose-inducible promoter (P_araB_-*fliC* ^T237C^ ∆*fliC*). Residue T237 in the variable loop of the FliC flagellin of *Salmonella enterica* was chosen for cysteine substitution because it is accessible for external labeling (Figure S1A). The motility of otherwise wild type *Salmonella* cells harboring the *fliC* ^T237C^ mutation was approximately 64% of the wild type *fliC* allele (Figure S1B).

**Figure 1:**
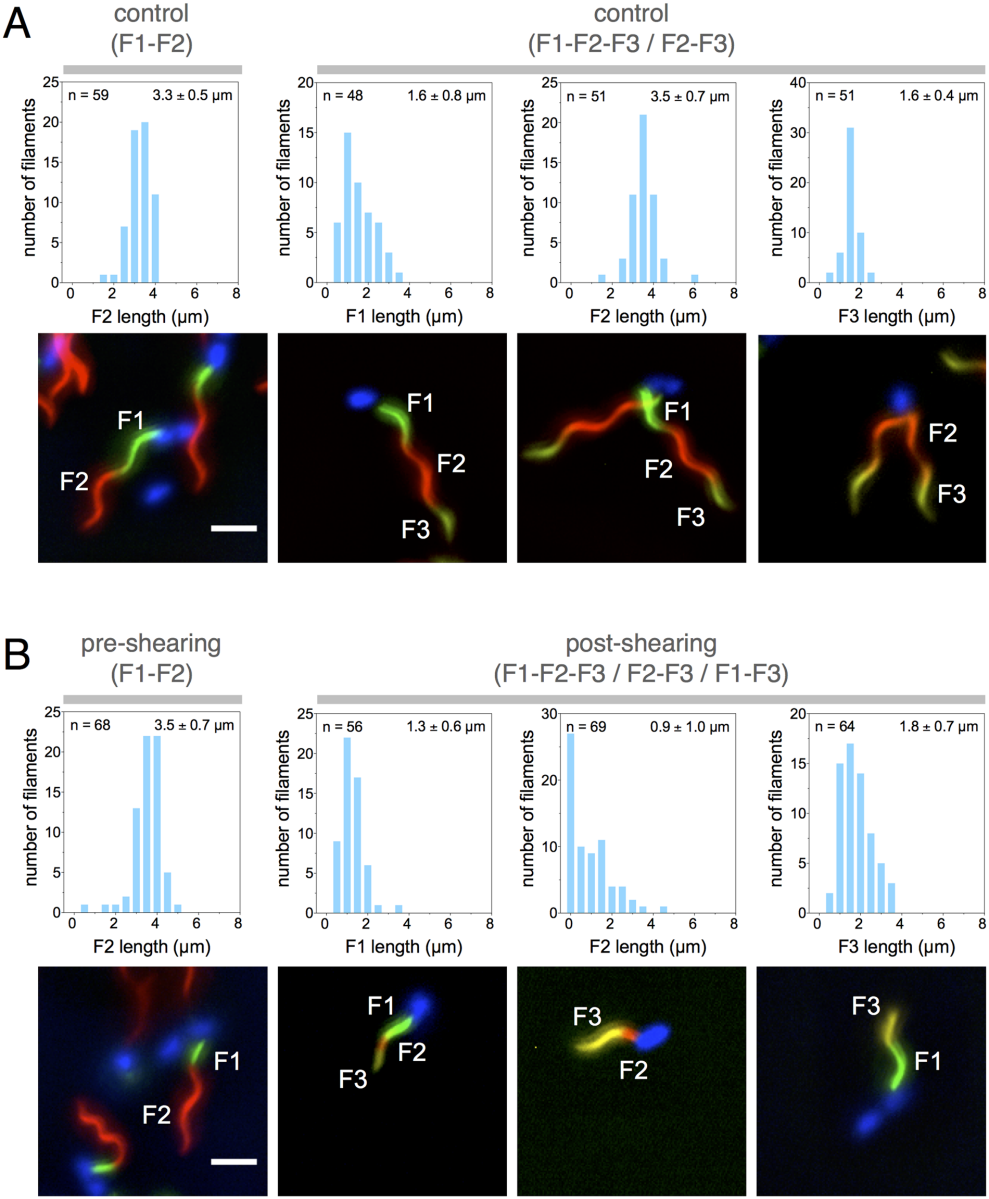
Broken flagella re-grow after mechanical damage. Strain EM4067 (P_*araBAD*_-*fliC* ^T237C^ ∆*fliC*) was used to assess re-growth of flagellar filaments after mechanical shearing using a 3-color *in situ* labeling protocol. (**A**) Top: F1, F2 and F3 filament fragment lengths of the control sample after 2-color labeling (left panel) and 3-color labeling (right panels). Bottom: Representative fluorescent microscopy images. Scale bar represents 2 µm. (**B**) Top: F1, F2 and F3 filament fragment lengths of the shearing sample after 2-color labeling (pre-shearing, left panel) and 3-color labeling (post-shearing, right panels). Bottom: Representative fluorescent microscopy images. Scale bar represents 2 µm.

After a pulse induction of the expression of *flhDC*, incubation of strain EM4067 was resumed in the absence of inducer to prevent formation of another round of basal-body complexes, which facilitated length measurements of the filament segments. Subsequently, expression of *fliC* ^T237C^ was induced from the chromosomal P_*araBAD*_ promoter in the presence of the first fluorophore coupled to a cysteine-specific maleimide moiety. This allowed for the simultaneous initiation of flagellar filament assembly and labeling of the first filament segment (F1). After 60 minutes of *in situ* labeling of the growing filaments, the first fluorophore was removed and labeling of filaments was resumed for an additional 60 minutes using a second fluorophore-coupled maleimide (F2, using a different color than F1). The filament segment F2 grew to an average length of approximately 3.5 µm. Afterwards, the flagellar filaments were mechanically sheared, followed by incubation in the presence of a third fluorophore-coupled maleimide (F3) for an additional 30 minutes. The control sample was manipulated the same way, except that filaments were not mechanically sheared. The 3-color labeling allowed us to determine if a filament stopped growing or had been broken by mechanical shearing forces. In case of the control sample, we observed only flagella labeled with a pattern of F2-F3 or F1-F2-F3 segments, which demonstrated that none of the flagella stopped growing or broke during the duration of the experiment (Fig. 1A).

For the sheared sample, we observed that 7% (5 out of 69) of the analyzed filaments were not labeled with the F3 fragment, indicating that the shearing event prevented re-growth. We considered only filaments that displayed a labeling pattern of F1-F3 (*i.e.* filaments, which lost the F2 segment) as filaments that had been broken, but re-grew after the shearing event. 41% of the observed filaments (26 out of remaining 64 filaments) displayed a F3 fragment with an average length of 1.8 ± 0.6 µm on top of a F1 fragment. This demonstrated that re-growth could occur after shearing (Fig. 1B). For the remaining 38 filaments, which retained a F2-F3 or F1-F2-F3 pattern, the pattern of filament segments does not unequivocally demonstrate that they were broken. However, the short length of the F2 fragment (average F2 length of 0.9 ± 1.0 µm) as compared to the length of the F2 fragment of the control sample and pre-shearing (average F2 length of 3.5 ± 0.7 µm) suggested that these filaments were also sheared and indeed re-grew during the F3 labeling step.

Broken flagellar filaments have lost their original filament cap and would need the ability to re-assembly a new capping structure before they are able to resume growth. We thus tested whether we could observe the formation of a new filament cap on top of previously sheared flagella. A hemagglutinin (HA) epitope tag was inserted in the cap plate accessible area of the filament cap FliD to facilitate visualization by immuno-gold electron microscopy^16, 17^. In a strain expressing *fliD*::HA from its natural promoter, approximately 90% of the flagellar caps were labeled with one to five gold nanoparticles. For approximately 10% of the observed flagella, the pentameric flagellar cap was associated with five gold particles (using 15 nm gold particles), indicating that the probability for the HA epitope tag to be accessible in the right plane and well labeled by the immunogold procedure was quite low.

Strain TH22231 harbored (i) the flagellar master regulatory operon under control of an AnTc inducible promoter (P_*tetA*_-*flhDC*) for synchronized production of flagellar basal bodies, (ii) the flagellin *fliC* ^T237C^ cysteine substitution at residue T237 in the variable loop of the FliC flagellin expressed from the chromosomal *fliC* locus for labeling of sheared filament segments, and (iii) the HA-epitope tagged *fliD* construct under control of an arabinose-inducible promoter (P_araBAD_-*fliD*::HA) in addition to the native *fliD* gene. The inducible HA-epitope tagged FliD construct allowed us to express FliD-HA at later stages of filament assembly and thus visualize formation of newly formed cap structures on the tip of broken filaments.

After a pulsed induction of *flhDC* expression, incubation of strain TH22231 was resumed in the absence of AnTc inducer to prevent formation of another round of basal-body gene expression. After 30 min incubation to allow for filament growth, 5 nm gold particles coupled to cysteine-specific maleimide moieties were added for additional 40 minutes to label the already assembled filaments *in situ*. Gold-labeling of the initially assembled filament allowed us to identify (basal) filament segments that were made prior to any shearing event. Excess gold maleimide was removed by mild centrifugation and the flagellar filaments were mechanically sheared as described above. Prior to mechanically shearing the flagella, we induced expression of HA-epitope tagged FliD by addition of arabinose in order to visualize newly formed cap structures. Since wild type *fliD* was constitutively expressed from its native locus during the experiment, both HA-epitope tagged FliD and wild type FliD were secreted simultaneously, and this reduced the probability to observe cap structures formed only by HA-epitope tagged FliD. The overproduction of arabinose-induced *fliD::*HA increased the probability of detecting the assembly of a new HA-epitope tagged FliD cap on the tip of a broken filament, however, the low frequency prevented an adequate quantitative analysis of the probability of re-growth and formation of new capping structures.

The filament structures were stained with methylcellulose-uranyl acetate and visualized by electron microscopy. The differential immunogold labeling (filaments before breakage were labeled with 5 nm gold particles and HA-epitope tagged FliD cap protein was labeled with 15 nm gold particles) thus allowed us to determine if a new HA-epitope tagged FliD cap assembled on top of previously broken filament or on top of a newly formed filament.

**Figure 2:**
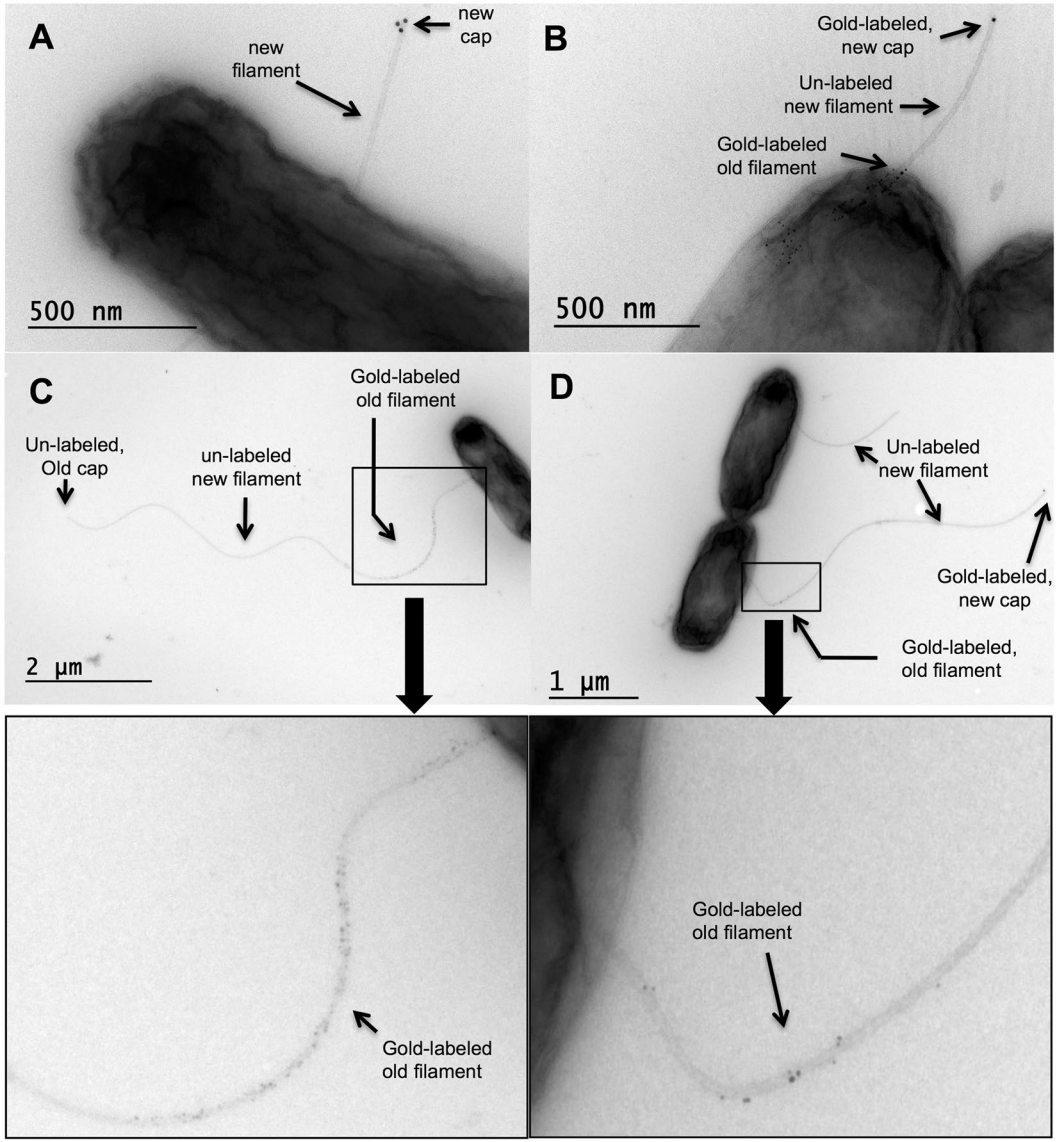
Formation of a new filament cap on re-grown flagellar filaments. (**A–D**) Representative electron microscopy images of gold-labeled flagellar filaments and newly formed FliD cap structures of strain TH22231 (P_*araBAD*_-*fliD*::HA *fliC* ^T237C^) demonstrating that broken filaments re-grow. Flagellar filaments were gold-labeled before mechanically shearing the flagella using viscous shearing forces. Induction of hemagglutinin (HA) epitope tag-labeled FliD from the P_*araBAD*_ promoter (indicated as ‘new cap’ in the figure) was induced after mechanical shearing of the filaments.

In the not-sheared control sample, we detected HA-epitope tagged FliD caps (using 15 nm gold particles) in approximately 40% of the newly formed filaments. These were nascent filaments that were not labeled with 5 nm gold-maleimide during the period of initial filament growth (Fig. 2A). We did not detect HA-epitope tagged FliD on the tip of any of the long filaments that displayed gold-labeled first filament segments, but continued to grow after the initial labeling with 5 nm gold-maleimide.

As expected, we observed shorter gold-labeled basal filament segments for flagella that were mechanically sheared compared to the gold-labeled basal filament segments of not-sheared control samples (Fig. 2). We considered flagellar filaments that displayed short, gold-labeled basal filament segments followed by an unlabeled filament segments as flagella that had successfully been sheared. Importantly, we observed HA-epitope tagged FliD caps on the tip of such previously sheared flagellar filaments (Fig. 2B,C). Thus, the detection of HA-epitope tagged FliD on the tip of previously broken and re-grown filaments unequivocally demonstrates that a new filament cap can reassemble to allow re-growth of broken flagella.

An alternate method to break filaments was then used in order to test the possibility that re-growth of flagellar filaments depends on how they were broken. Ultrashort laser pulses are frequently used to manipulate biological tissues^18–21^. Here, we focused laser pulses of 75 fs duration (centered at a wavelength of 780 nm) on our sample with a 100 × 1.3 NA microscope objective. A wavelength between 700–1100 nm was used since water and most biological tissues are transparent in this interval^20^. Under these conditions, no heat is accumulated at the focal point because the heat diffusion time (~98% of the heat is dissipated after 1 µs) is much smaller than the time between two pulses (greater than 4 µs)^18, 20^. Physical damage to the material is not thermal, but rather induced by the emission of a shock wave driven by the rapid expansion of a laser-induced plasma (a so-called Coulomb explosion)^22, 23^. In other words, the use of ultrashort pulses implies that very little energy is deposited (essentially no heat) and that the material near its focal point is damaged by the creation of a very localized shock wave. That shock wave was used to break individual flagellar filaments placed in the vicinity of the laser beam.

**Figure 3:**
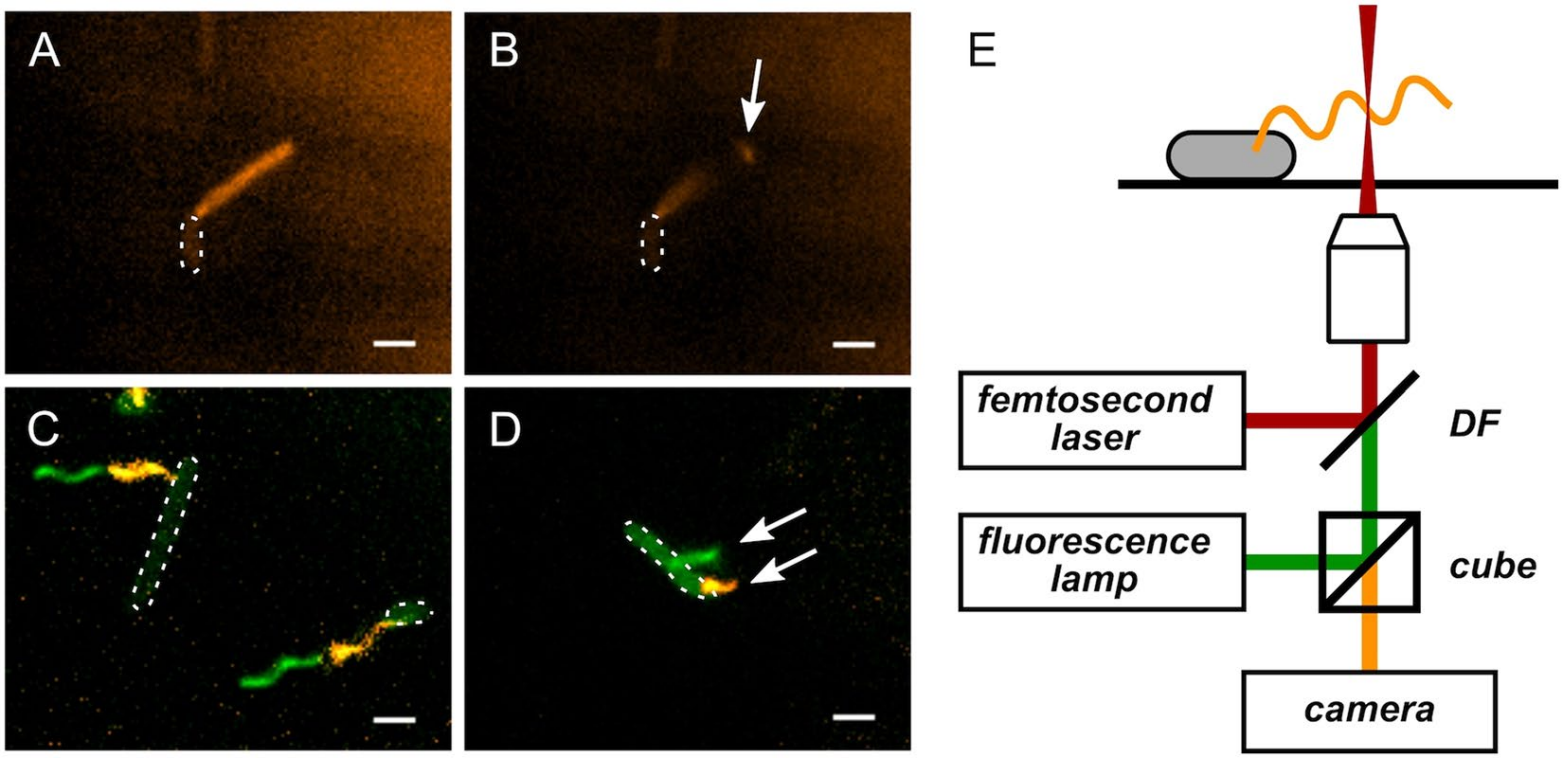
Flagellar filaments broken using ultrashort laser pulses do not re-grow. (**A,B**) Flagellar filament of strain EM800 (Δ*fliO* Δ*flgM* PflhD* fliC ^T237C^) before (**A**) and after (**B**) being broken by an ultrafast laser beam. The cell body is barely visible (highlighted with white dotted line) and the filament shows up large and fuzzy because it is rotating much faster than the image acquisition rate. The white arrow points to the broken filament segment drifting away and out of focus. Scale bars are 2 µm. The full movie is available in Supplementary Materials. (**C**) Control cells of strain EM800 whose filaments were left intact after 2-color labeling. The green portions of filaments that grew during incubation are clearly distinguishable. (**D**) Example of a bacterium (EM800) that grew a new flagellum during incubation. The top arrow points to the new filament that grew after the first labeling. The filament is blurry since it was rotating during the exposition. The bottom arrow points to the broken filament (orange) that did not regrow. The continued rotation of the flagellar filament demonstrates that the cell was still alive and potentially able to re-synthesize a new filament. (**E**) Schematic of the experimental setup. The femtosecond laser is added to the optical axis through a dichroic filter (DF) and focused on the sample with a 100 × 1.3 NA objective. The same objective is used for fluorescence imaging. The sample is illuminated with a broadband light source and a fluorescence cube selects the excitation and emission wavelengths. The bacterial filaments are then visualized using an EMCCD camera.

In order to break individual flagellar filaments and observe their re-growth, we needed an experimental setup that allowed for the unambiguous identification of individual filaments on a microscope slide over multiple hours. That was greatly simplified by using a bacterial strain for which the majority of cells possessed on average a single filament. A serendipitous discovery was made that a strain deleted for the *fliO* gene, harboring a P_*flhDC*_ P1 and P4 promoter up mutation^24^ and in addition missing the anti-σ^28^ factor FlgM (termed ΔfliO*) preferentially assembled only a single filament (Figure S1). The FliO component of the flagellar-specific type-III secretion apparatus is essential for export apparatus function and a ∆*fliO* strain is non-flagellated under normal export substrate conditions^25, 26^. However, it was recently reported that the requirement for *fliO* could be bypassed by mutations in *fliP* ^27^. We found that the ΔfliO* strain retained slight motility in soft-agar plates (Figure S1C) and that more than half of the cells of the ΔfliO* strain produced at least one flagellum (Figure S1D,E). We next introduced the *fliC*(T237C) substitution allele into a ΔfliO* strain to allow observation of flagellar filaments by fluorescent microscopy as described above. Cells of the ΔfliO* fliC(T237C) strain were immobilized in a custom-made flow chamber and labeled with Alexa Fluor® 546 coupled to maleimide. Only cells that were firmly attached to the coverslip, and that displayed a single flagellum were selected for laser damage of the filament. In order to ensure that the observed cell was alive and healthy, we considered only cells with rotating filaments. In addition, we selected filaments that were not only rotating on their axis, but also slowly gyrating (*i.e*. the filament axis itself was rotating around slowly). Indeed, initial trials showed that if the filament was not gyrating, the laser pulses which broke the filament frequently stopped the rotation of the motor. It is not exactly clear why that was the case, but presumably non-gyrating filaments have a much stronger tendency to stick to the cell body or the coverslip when shortened. The ideal candidate was therefore a rotating filament that was also gyrating in a somewhat uniform circular trajectory. The laser beam was then positioned in the vicinity of the bacterium (about 2 or 3 µm away from the cell body) so that its filament would break by moving through the ultrafast laser pulses. A successful filament breakage was clearly identified by the acceleration of the filament gyration, and the broken filament fragment was often seen diffusing away. To establish that the laser did not damage the flagellar motor or compromise the cell membrane, we made sure that the broken filament was still rotating. Figure 3 shows the same bacterium before (panel A) and after (panel B) its filament was broken. The length of the broken filament was reduced from ~3.5 µm to ~2 µm. The full movie is available in the Supplemental Material online (Movie S1).

A total of 62 individual bacterial filaments were broken using the femtosecond laser and re-visited after a two-hour incubation period. Filament re-growth was not observed on any of the filaments that had been broken. Statistically, the proportion of filaments that can re-grow after being broken by the laser pulses is less than 5% (by the “rule of three”, 3/*n* = 3/62 = 5% with 95% confidence, Table 1)^28^. The fact that a filament was still rotating after incubation demonstrates that this particular bacterium was alive and healthy (and therefore potentially able to re-grow filaments). However, if a filament lacked motion, it was most likely because it simply stuck to the surface of the poly-L-lysine-coated coverslip. We included such filaments in Table 1 because dead bacteria were easily identified due to their cell bodies filling up with fluorophore. As a control, we observed many filaments that were left intact on the same coverslip. As shown in Fig. 3C, the portion of the filament that grew during incubation is clearly visible as a green extension at the end of the orange filament (when images in both channels are combined digitally). To acquire such image, the filament’s rotation had to be stopped either by exposing the bacterium to a large amount of blue-green light, or by punching a hole in the cell body with the laser. Over 90% of the unbroken, but rotating filaments displayed a distal “green” filament segment after the incubation period, which demonstrates that they continued to grow.

**Table 1:** Number and re-growth of filaments broken using an ultra-fast laser pulse.

Strain	Total	Still turning	Stopped
EM800	44	16 (36%)	28
EM1283	18	8 (44%)	10

The rotation status of the filaments when we revisited them is also detailed. None of these 62 filaments continued to grow after being broken.

Bacteria that grew a new, second filament on the same cell during the second labeling period were occasionally observed. Figure 3D shows a cell on which the new filament (green and fuzzy due to rotation) is seen besides the old broken filament (orange) that clearly did not regrow. Such cases with “built-in” control further support our conclusion that filaments do not re-grow after being broken by the laser.

The range of laser parameters explored was limited by the available technology and practical considerations. The laser wavelength was fixed around 780 nm, but because the shock wave is highly non-linear, small variations in the laser wavelength should not play a significant role^29^. A wavelength between 700–1100 nm was used since water and most biological tissues are transparent in that region^20^. In terms of laser power, we used the minimum pulse energy that could break filaments (around 0.5 nJ/pulse). With higher pulse energy, the cell body (a few micrometers away from the focus) would sometimes be pushed, and we needed the body to stay firmly attached to the coverslip to be able to re-visit it a few hours later (after a number of washing operations). For the same reason, we could not break the filament too close to the cell body. Finally, most of the filaments were broken with a laser repetition rate of 1 kHz, but some experiments were also performed at 250 kHz. As expected, that did not change the outcome of the experiments (no re-growth) because a filament can be broken with a single pulse, and not the accumulation of energy from many pulses.

The ΔfliO* strain EM800 lacks the anti-σ^28^ factor FlgM, which ensures constant σ^28^-dependent gene expression from Class 3 promoters. Accordingly, both flagellin subunits and the filament cap FliD should be expressed and available for filament re-growth. As shown in Fig. 3D, EM800 cells re-grew new flagella following damage of the initial filament by the laser. In order to provide an excess of cap protein, we also performed the laser shearing experiments with a strain that overexpressed *fliD* from an inducible arabinose promoter (EM1283, see Figure S2). This enabled us to test the possibility that excess FliD in the cytoplasm could accelerate the formation of a new cap structure, and thereby allow filament growth after damage. We performed the same manipulations with strain EM1283 and, as outlined in Table 1, none of the 18 damaged filaments grew back (44% were still turning after incubation), while undamaged two-color filaments were frequently observed. Figure S2A shows complementation of a Δ*fliD* strain by overexpressed *fliD* in a motility plate assay. The same arabinose-inducible *fliD* construct did not affect motility of the ΔfliO* strain EM1283.

## Discussion

It has always been assumed that flagella, which easily break when exposed to shear forces, are able to re-grow. However, experiments that demonstrated motility recovery after shearing did not distinguish actual re-growth of sheared flagella from continued growth of nascent flagella that were not sheared. This work examined the possibility that a flagellar filament of *Salmonella enterica* could continue to grow after breakage. We used two methods to break flagellar filaments: (i) the traditional method of mechanical shearing and (ii) exposure of the filament to femtosecond laser pulses. The combination of femtosecond laser and the use of a single-filament bacterial strain enabled us to achieve the technical challenge of inducing specific damage to identified filaments and re-visit individual bacteria after an incubation period.

The results of our experiments are unequivocal and show that the ability of a bacterial filament to re-grow depends on how it was broken. We observed re-growth of filaments after breakage by viscous shearing. We improved previous dual-color filament labeling approaches by additional labeling with a third color, which allowed us to distinguish re-growth on previously broken flagella from continued growth of filaments that did not break or flagella that newly grew (Fig. 1). We further used differential electron microscopy to detect newly formed filament cap structures on previously broken and re-grown filaments (Fig. 2). Filaments broken by the laser, however, did not re-grow (Fig. 3).

The process of flagellar assembly and its genetics has been extensively studied^1, 2, 30, 31^. A crucial structure in the specific question studied here is the filament cap, which is a protein complex at the distal end of the flagellar filament^8, 17^. The principal role of the filament cap is to allow the flagellin proteins to polymerize at the tip. The flagellin proteins are synthesized in the cytoplasm of the cell and exported via the flagellum-specific type-III secretion system before they travel diffusively through the central channel of the flagellum^6^. The cap structure is formed by a protein called FliD (or hook-associated protein HAP2), and is essential for the growth of filaments^32, 33^. The observation that hook-associated proteins are constantly secreted through the filament channel suggested that a lost cap could be replaced^30^. On the other hand, Homma and Iino concluded that the FlgL hook-associated protein (HAP3) was essential to the attachment and assembly of the cap^34^. FlgL is located at the base of the flagellum between FlgK (HAP1) and the beginning of the filament. A filament that broke at the base might not possess the FlgL interface, which would result in continuous secretion of cap proteins that do not polymerize at the tip of broken structures. Without a cap, the broken filament would not be able to re-grow. Filaments broken past the FlgKL-flagellin interface at the flagellar base would potentially allow for re-growth. In the present study, we now visualize – for the first time – a newly formed cap on top of a previously broken and re-grown filament (Fig. 2). Thus, the FliD cap can re-assemble also on broken filaments that only possess the flagellin interface.

We next addressed whether the method used to shear filaments had an impact on the ability of the filaments to re-grow or not. As demonstrated in Figs 1 and 2, mechanically sheared filaments (by passage through a small syringe needle) continued to grow whereas filaments sheared with ultrashort laser pulses did not. Intuitively, one could be tempted to think that the heat deposited by the laser pulse “cauterized” the end of the filament and thus prevented further growth. In our experiments the bacterial filaments slowly approach the focal point of the laser, from which a shock wave was emitted every millisecond (or more). The energy contained in each laser pulse (about 5 × 10^−10^ J) would be sufficient to break the hydrogen bonds between the protein subunits constituting the filament (~20 kJ/mol = 3 × 10^−20^ J/bond), or even the peptide bonds inside those proteins (~300 kJ/mol = 5 × 10^−19^ J/bond)^35^. It thus appears possible that laser pulses might damage and destroy individual flagellin proteins. Presumably, damaged or unfolded flagellin proteins left at the tip of the filament could interfere with the assembly of a new cap. However, the possibility that intact flagellin proteins would be left at end of the filament might also be reasonable since the binding energy between FliC proteins (hydrogen bonds) is lower than the energy of the peptide bonds within the protein (at least 1/10^th^), and also because the filament approaches very slowly the focal point of the laser from which a shock wave is emitted regularly. If the laser does not “cauterize” the filament, what else could explain the difference between laser-shearing and mechanical shearing ? Even though this is highly speculative, we would like to propose the hypothesis that when a filament is mechanically sheared by passage though the tip of a small syringe needle, mechanical forces are applied on it, which might induce polymorphic transitions just before it breaks (for example into a “straight” form as observed previously^36^). This could possibly lead to a broken tip with a surface on which a cap could reform, thereby enabling growth. The shock of breakage exerted on the filament is certainly more sudden and local in the case of laser-shearing and might explain why breaking filaments with ultrashort laser pulses prevents the formation of a new cap.

In conclusion, we observed broken flagellar filaments could regrow and that the method used to break bacterial filaments has an impact on whether a broken filament will re-grow or not. This surprising result once again highlights the richness of biophysical studies at the individual cell level, probing the mechanical properties of protein assemblies and the impact of these properties on biological function.

## Materials and Methods

### Bacterial strains and growth conditions

The *Salmonella enterica* serovar Typhimurium (*Salmonella* Typhimurium) strains used in this study are listed in Table 2. The generalized transducing phage of *Salmonella* Typhimurium P22 *HT105/1 int-201* was used in all transductional crosses^37^. Strains were streaked for single colonies from frozen stock (−80 °C) on lysogeny broth (LB) plates (10 g Bacto tryptone, 15 g Bacto agar and 5 g NaCl per liter). For the laser-breakage experiments, an isolated colony was inoculated in 10 ml TB broth (10 g Bacto tryptone and 5 g NaCl per liter) in 125 ml Erlenmeyer flask and placed at 34 °C for 15 hours with gyration at 200 rpm. A volume of 100 µl of the saturated culture was inoculated in 10 ml TB broth in 125 ml Erlenmeyer flask and placed at 34 °C for 4 hours with gyration at 200 rpm, until it reached an OD_600_ ≈ 0.45 *(~*4 × 10^8^ cells/ml*)*. The culture was centrifuged for 5 minutes at 1,500 × *g* and gently resuspended in 1 ml motility buffer (MB) (0.01 M potassium phosphate at pH 7.0, 10^−4^ M EDTA). For quantitative assessment of motility in soft-agar plates, single colonies grown overnight on a LB plate were inoculated in soft-agar motility plates (10 g Bacto tryptone, 3 g Bacto agar and 5 g NaCl per liter) for 4.5 hours at 37 °C. The diameter of the motility swarms was measured using ImageJ^38^, and the motility relative to a wild type control was calculated.

**Table 2:** *Salmonella enterica* serovar Typhimurium LT2 strains used in this study.

Strain number	Relevant genotype	Reference
TH6232	Δ*hin-5717*::FRT	Lab collection
TH9671	Δ*hin*-*5717*::FRT *fliC6500*(T237C)	Lab collection
TH10548	Δ*fliO6708*(Δaa6–121)	Lab collection
TH16123	Δ*flgM5628*::FRT Δ*fliO6708* P*flhDC7460* Δ*hin*-*5717*::FCF	This study
EM800	Δ*flgM5628*::FRT Δ*fliO6708* P*flhDC7460* Δ*hin*-*5717*::FCF *fliC6500*(T237C)	This study
EM808	Δ*araBAD1005*::FRT	This study
EM1283	Δ*flgM5628*::FRT Δ*fliO6708* Δ*hin-5717*::FCF *fliC6500*(T237C) Δ*araBAD980*::*fliD*+	This study
EM1730	Δ*flgM5628*::FRT Δ*fliO6708* Δ*hin*-*5717*::FCF *fliC6500*(T237C) Δ*araBAD1005*::FRT	This study
EM1769	Δ*fliD5630*::FRT Δ*araBAD980*::*fliD* ^+^	This study
EM4067	Δ*hin-5717*::FRT ∆*araBAD1209*::*fliC*(T237C) ∆*fliC7861*::FRT P*flhDC5451*::Tn10dTc[del-25]	This study
TH22231	Δ*hin*-*5717*::FRT *fliC6500*(T237C) P*flhDC5451*::Tn10dTc[del-25] Δ*araBAD2023*::*fliD*::HA	This study

### Flagellin labeling and fluorescent microscopy

A custom-made flow-cell was fabricated using a standard microscope slides (25 × 75 mm) and an 18 × 18 mm coverslip (cleaned with 70% ethanol). Two stripes of Parafilm were placed ~1 cm apart between the slide and coverslip and gently pressed after heating over a flame. A drop of Poly-L-lysine 0.01% (Sigma) was left on the coverslip for 5 minutes and then rinsed before assembly. This tunnel was then filled with 50 µl of cell suspension and left 10 minutes upside-down for the cells to deposit and stick to the coverslip. Cells that were still in suspension were rinsed away with motility buffer (MB). To label the filaments, the flow-cell was then filled with 50 µl of Alexa-Fluor maleimide 546 dye (A-10258, Life Technologies) at 1 mM concentration and the flow-cell was left in a dark humidity chamber for 1 hour at room temperature. The excess dye was washed by gently flowing 600 µl of MB, and the filaments could then be observed in fluorescence microscopy. After breaking filaments with the laser (as described below), a second labeling was performed with a different dye: Alexa-Fluor maleimide 488 (A-10254, Life Technologies). During that second labeling, the dye was diluted (again at 1 mM) in TB and the cell was left in the dark humidity chamber at 37 °C for 2 hours. For overexpression of the FliD protein from the inducible arabinose promoter, arabinose was added to the TB broth during the second labeling to a final concentration of 0.2%. These observations were performed under an IX71 microscope from Olympus (100 × 1.3NA objective) using an Excite light source (EXFO) and images were processed in Matlab.

Alternatively, flagellar filaments of FliC-locked strains were labeled using anti-FliC immunostaining as described previously^39^. Images were collected using an inverted Applied Precision Deltavision microscope and assembled using ImageJ.

### Laser shearing

Between the two labeling steps, individual flagellar filaments were broken using ultrashort laser pulses. The laser source used in these experiments was a RegA 9000 (Coherent) providing ultrashort pulses (~75 fs duration) centered on a wavelength of 780 nm at a repetition rate of 1 or 250 kHz. The energy of the pulses entering the microscope was controlled using a motorized rotating half wave retardation wave-plate placed between two crossed polarizers. The optical power at the entrance of the microscope was estimated to be 120 µW (or 0.48 nJ/pulse). As shown in Fig. 3, the laser was focused on the sample by the same 1.3 NA objective used for imaging. After a filament was broken, the position of its bacterium was logged by noting the coordinates of the 3-axis micro-manipulator (MP-285, Sutter Instruments) that holds the microscope slide. High-speed videos of the bacterium (1 second at 500 frames per second) were recorded both in fluorescence and in bright field microscopy with a EMCCD camera (iXon 888, Andor Technology). After breaking ~10 filaments (generally ~1 h), the slide was placed at 37 °C for the “regrowth” period (and second labeling). To account for the small variations in the position of the slide on the holder, the position of two reference points were recorded at the beginning of the manipulations. After the 2 h incubation period, the coordinates of the two reference points were noted again and the new coordinates of the bacteria of interest were calculated. Each one was then revisited and videos at the wavelength of both fluorophores were recorded.

### Flagellar labeling and mechanical shearing of flagellar filaments

An isolated colony of the strain of interest (EM4067 or TH22231) was inoculated overnight in LB or TB broth at 37 °C. A 1:100 dilution of the saturated culture was then inoculated in 25 ml in a 125 ml Erlenmeyer flask and placed at 30 °C for 2 h with gyration at 200 rpm. Anhydrotetracycline (AnTc) was then added at a final concentration of 100 ng/ml to trigger basal bodies production by inducing expression of the flagellar master operon *flhDC*. After 30 min, the culture was centrifuged for 2 min at 2,500 × *g* and gently resuspended in PBS buffer to remove AnTc. Cells were centrifuged for 2 min at 2,500 × *g* and gently re-suspended into the media and grown for an additional 30 min before induction of *fliC* transcription from the P_*araBAD*_ promoter in case of EM4067 by addition of 0.2% arabinose. A 1 ml portion of cells was transferred into a 13 ml glass tube in the presence of the first conjugated maleimide dye (adding 1 µl of maleimide conjugated to Alexa Fluor 488 or DyLight-488 at a concentration of 10 mM (ThermoFisher Scientific); or 100 µl of freshly prepared reaction buffer with one vial of 5 nm maleimide-activated gold nanoparticles (Cytodiagnostic, CA)). The cells were grown for 40 to 60 min at 30 °C in presence of the maleimide conjugate. Cells were then centrifuged for 2 min at 2,500 × *g* to eliminate the first conjugate and gently resuspended in growth media. For the fluorescent labeling experiment, a second maleimide conjugated to Dylight-550 or Alexa Fluor 495 (ThermoFisher Scientific) was added for 1 h at 30 °C and the cells centrifuged again to eliminate the fluorescent dye. Cells were re-suspended in culture medium (0.2% arabinose was added in the case of the differential electron microscopy analysis to induce expression of *fliD::HA* from the P_*araBAD*_ promoter) and split in two Eppendorf tubes with 500 µl sample each. One of the samples was mechanically sheared by passing it 20 times in and out of a 1 ml syringe with a 27.5-gauge needle. The samples (including the non-sheared sample) were spun down for 2 min at 2,500 × *g* to eliminate broken filaments. Cells were re-suspended into 500 µl of growth medium containing 0.2% arabinose and incubated at 30 °C for 30 to 45 min in the presence of DyLight 650–4xPEG maleimide (ThermoFisher Scientific) in the case of the 3-color fluorescent labeling experiment. Cells were centrifuged for 2 min at 2,500 × *g* to eliminate the third fluorescent dye or to concentrate the cells.

### Imaging of 3 color-segment flagella by fluorescence microscopy

After the final maleimide-dye labeling, the bacteria were resuspended in PBS and applied to a custom-made flow cell made of a coverslip attached to a Polysine™ microscope slide (ThermoFisher) by two layers of double-sided sticky tape. After addition of PBS to flush non-adhering cells, the bacteria were fixed by addition of 2% formaldehyde and 0.2% glutaraldehyde in PBS for 5 min. Fluoroshield™ mounting medium containing DAPI (4′,6-diamidino-2-phenylindole) (Sigma-Aldrich) was added. For fluorescent microscopy, a Zeiss Axio Observer microscope at 100 × magnification was used and fluorescence images were analyzed using ImageJ.

### Imaging of the flagellar cap by immuno-gold electron microscopy analysis

After mechanical shearing, expression of *fliD*::HA and regrowth of flagellar filaments, cells TH22231 were fixed in 0.2% glutaraldehyde and 2% paraformaldehyde in Life cell imaging solution (Molecular Probes). To avoid further shearing, cells in fixative were then transferred onto a clean parafilm surface, not by pipetting, but with a stainless steel loop. This resulted in a sample drop of about 10 µl. A Formvar carbon-coated grid was then inverted onto this drop for cells to adhere. Immunogold labeling was carried out by floating the grid on sequential drops of solutions, as described^40^, using an HA tag monoclonal primary antibody (Thermofisher) at a dilution of 1:200 and a 15 nm gold conjugated Goat anti-mouse IgG(H + L) secondary antibody (Ted Pella, INC) at a 1:60 dilution. At the end of the labeling, cells were contrast-stained with methylcellulose-uranyl acetate (1.7%/0.43%). Air-dried samples were then examined with a transmission electron microscope (JOEL JEM-1400) operated at 120 kV.

## Electronic supplementary material

Supplementary Movie 1

Supplementary Material
