# Supplementary material for: Variability in bacterial flagella re-growth patterns after breakage
Source: Sci Rep. 2017 Apr 28;7:1282. doi: 10.1038/s41598-017-01302-5 (PMC5430758; doi:10.1038/s41598-017-01302-5)
Supplement: Supplementary file 2 — Supplementary Material [file 41598_2017_1302_MOESM2_ESM.pdf]

Supplementary Material

Variability in bacterial flagella re-growth patterns after breakage

Guillaume Paradis[a,†], Fabienne F. V. Chevance[b,†], Willisa Liou[b], Thibaud T. Renault[c], Kelly T. Hughes[b], Simon Rainville[a,*] and Marc Erhardt[c,*]

[a]Department of Physics, Engineering Physics and Optics and Centre of Optics, Photonics and Lasers, Laval University, Quebec City, Quebec, Canada

[b]Department of Biology, University of Utah, Salt Lake City, Utah, 84112, USA

[c]Helmholtz Centre for Infection Research, 38124 Braunschweig, Germany

[†]Co-first authors

[*]Co-senior authors and corresponding authors

Correspondence:

- Simon Rainville; Department of Physics, Engineering Physics and Optics; 2375, rue de la Terrasse; Quebec City (Quebec); Canada G1V0A6; Tel: 418-656-2131 ext 12511; simon.rainville@phy.ulaval.ca

- Marc Erhardt; Helmholtz Centre for Infection Research, 38124 Braunschweig, Germany; Tel: +49-531-6181-4800; marc.erhardt@helmholtz-hzi.de

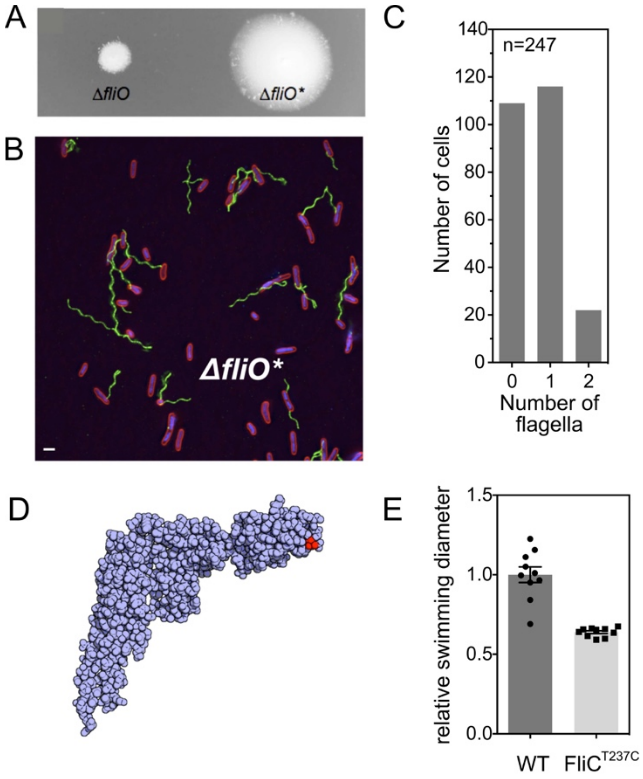

Supplementary Figure 1: Motility and flagellation of single-flagellum strain TH16123. (A) Enhanced motility of strain TH16123 that is deleted for *fliO* and that has increased flagellar gene expression resulting from deletion of the negative-regulator FlgM and a promoter-up mutation in the *flhDC* operon (Δ*fliO**). Motility plates were incubated overnight for 18 hours before imaging. The parental strain TH10548 deleted for *fliO* displays a non-motile phenotype. (B) Fluorescent microscopy analysis revealed the preferential formation of a single flagellum in the Δ*fliO** strain TH16123. Representative fluorescent microscopy image of the Δ*fliO** strain. Flagellin FliC was immunostained as described in Materials and Methods. Membranes were stained using FM-64 and DNA using DAPI. Scale bar 2 μm. (C) Quantification of numbers of flagella per cell of the Δ*fliO** strain by anti-FliC immunostaining. (D) Graphical visualization of the surface localization of the cysteine-substituted residue T237 (shown in red) using PDB no. 1IO1 of flagellin. (E) Relative swimming motility of strain TH9671 harboring a T237C substitution in the flagellin FliC compared to the wild-type control TH6232. Swimming motility was assayed using soft-agar swimming plates containing 0.3 % agar.

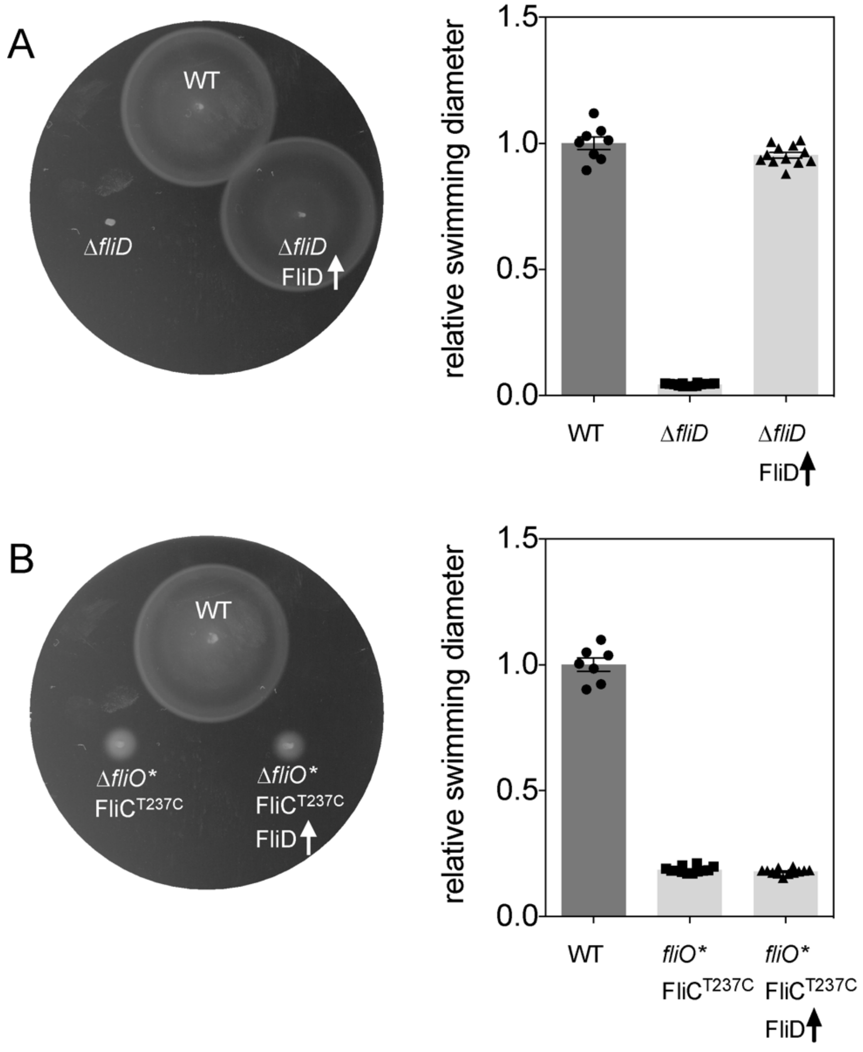

Supplementary Figure 2: Functional analysis of arabinose inducible *fliD*. (A) Left panel: Exemplary motility plate of strains EM808 (WT), EM1770 (Δ*fliD*) and EM1769 (Δ*fliD* P$_{araBAD}$-*fliD*$^{+}$) in the presence of 0.2 % arabinose. Right panel: Quantification of motility of a Δ*fliD* complemented by arabinose-inducible *fliD*. (B) Overexpression of *fliD* does not affect motility of the Δ*fliO** strain. Left panel: Exemplary motility plate of strains EM808 (WT), EM1730 (Δ*fliO**) and EM1283 (Δ*fliO** P$_{araBAD}$-*fliD*$^{+}$) in the presence of 0.2 % arabinose. Right panel: Quantification of motility of the Δ*fliO** strains after overproduction of *fliD*.

Supplementary Movie 1: Flagellar filament of strain EM800 cut by the laser beam. The laser's position is fixed at the right of the cell body. The filament turns rapidly on itself (fuzzy because the frame rate is small, 30 Hz) and the axis of rotation also gyrates around slowly. When the filament first crosses the laser beam, it is not cut but the fluorophores near the focal spot photobleach. On the second pass, the filament is cut and the free end can be seen diffusing away. Also note the acceleration of the gyration speed because the filament is now shorter.
